# COLOMBOS v3.0: leveraging gene expression compendia for cross-species analyses

**DOI:** 10.1093/nar/gkv1251

**Published:** 2015-11-19

**Authors:** Marco Moretto, Paolo Sonego, Nicolas Dierckxsens, Matteo Brilli, Luca Bianco, Daniela Ledezma-Tejeida, Socorro Gama-Castro, Marco Galardini, Chiara Romualdi, Kris Laukens, Julio Collado-Vides, Pieter Meysman, Kristof Engelen

**Affiliations:** 1 Department of Computational Biology, Research and Innovation Center, Fondazione Edmund Mach, San Michele all'Adige, Trento (TN) 38010, Italy; 2 Department of Biology, University of Padova, Padova (PD) 35121, Italy; 3 Interuniversity Institute of Bioinformatics Brussels (IB^2^), ULB-VUB, Triomflaan CP 263, B-1050 Brussels, Belgium; 4 Department of Genomics and Biology of Fruit Crops, Research and Innovation Centre, Fondazione Edmund Mach, San Michele all’ Adige, Trento (TN) 38010, Italy; 5 Programa de Genómica Computacional, Centro de Ciencias Genómicas, Universidad Nacional Autónoma de México, Cuernavaca, Morelos 62210, Mexico; 6 EMBL-EBI, Wellcome Trust Genome Campus, Cambridge CB10 1SD, UK; 7 Department of Mathematics and Computer Science, University of Antwerp, B-2020 Antwerp, Belgium; 8 Biomedical Informatics Research Center Antwerp (biomina), University of Antwerp/Antwerp University Hospital, B-2650 Edegem, Belgium

## Abstract

COLOMBOS is a database that integrates publicly available transcriptomics data for several prokaryotic model organisms. Compared to the previous version it has more than doubled in size, both in terms of species and data available. The manually curated condition annotation has been overhauled as well, giving more complete information about samples’ experimental conditions and their differences. Functionality-wise cross-species analyses now enable users to analyse expression data for all species simultaneously, and identify candidate genes with evolutionary conserved expression behaviour. All the expression-based query tools have undergone a substantial improvement, overcoming the limit of enforced co-expression data retrieval and instead enabling the return of more complex patterns of expression behaviour. COLOMBOS is freely available through a web application at http://colombos.net/. The complete database is also accessible via REST API or downloadable as tab-delimited text files.

## INTRODUCTION

COLOMBOS is a collection of expression data from both microarray and RNA-Seq experiments for several prokaryotic species, taken from publicly available database such as the Gene Expression Omnibus (GEO) (1) and ArrayExpress (2). Its uniqueness resides in the ability to cope with data heterogeneity and directly integrate data coming from different platforms and technologies. Other gene expression compendia are usually built either from data for a single transcriptomics platform or they rely on the integration of expression analysis results, rather than the integration of the actual measurements. In COLOMBOS however, data are collected and curated starting from the original raw intensities for microarrays and sequence reads for RNA-Seq, and then processed with a robust normalization and quality control pipeline to allow direct comparison of gene expression behaviour across different experiments and platforms (3). This results in a single expression matrix for every species, its rows representing the measured genes and its columns representing condition contrasts, comparisons between test and reference samples of different biological conditions. Attention is also given to the acquisition of meta-data related to the description of the biological conditions surveyed in an experiment, so that all the included samples and condition contrasts are formally annotated by means of a controlled vocabulary of condition properties. This annotation is a manual effort with the purpose of making the data comparable from a biological viewpoint and to yield reliable interpretations of expression patterns.

COLOMBOS compendia are accessible using the web interface, through a set of REST API calls, or via the R (4) package Rcolombos; they are also available for download in their entirety for use of COLOMBOS data in third-party stand-alone applications. Different types of analyses can be done using the COLOMBOS web interface itself; typical operations include starting from a set of known genes to find the conditions where they are (co)-expressed or to identify additional co-expressed genes. COLOMBOS’ tools are designed for users to ‘play around’ with the compendia, exploring the data with respect to the biological question they are interested in. They are encouraged to try different types of search queries based on genes or conditions, the available annotations or by relying on the actual expression values in a way reminiscent of a BLAST functionality with gene expression behaviour instead of sequence similarity. They can then visualize their results, use them as a basis for new queries to find additional (anti-)co-expressed genes, generate clusters to separate disjoint expression profiles, explore the overlap between multiple query results and potentially combine them, etc. There are several detailed use case tutorials on the website, illustrating step-by-step how concrete examples of conceptually different biological questions could be handled through the COLOMBOS interface. The previous v2.0, with all of its original databases and tools, will be kept available for future reference alongside COLOMBOS v3.0; how to access it is explained in the website's Help section.

## DATA CONTENT UPDATE

COLOMBOS v2.0 (5) was composed of seven bacterial species, four more than it contained at its inception. The current update includes an additional twelve species of biomedical or industrial relevance, including some Archaea. The main criteria for selecting these new species were the amount of publicly available expression data and quality of genome annotation and their perceived status as model organisms. A complete overview of the available species and associated statistics can be found in Table 1. The previous compendia have also been updated with recent experiments, in some extreme cases leading to an almost 2-fold increase of available data. For instance, the biggest compendium is that for *Escherichia coli*, which now contains over 4000 condition contrasts, nearly 2000 more than COLOMBOS v2.0 and almost as many as its number of genes, rendering the expression matrix virtually square. Gene lists, representing the species’ measurable transcripts, have been created from the NCBI RefSeq database (6) and various gene annotation data were added (or updated) from UniProt-GOA (7), RegulonDB (8), BioCyc (9) and EcoCyc (10), or species-specific published datasets (11).

**Table 1.** Overview of the data available in COLOMBOS

	Strain	Number of genes	Number of contrasts	Missing values (%)	First inclusion	Samples	Experiments	Platforms
*Escherichia coli*	MG1655	4321	4077	3.6	v1.0	5510	254 [15]	73
*Bacillus subtilis*	168	4176	1259	3.7	v1.0	1814	45	35
*Salmonella enterica serovar Typhimurium*	cross-strain	6261	1066	41.6	v1.0	1856	36	22
	LT2	4556	172	6.4	v2.0	316	8	10
	14028S	5416	681	22.7	v2.0	1252	17	7
	SL1344	4655	213	9.8	v2.0	288	11	9
*Streptomyces coelicolor*	A3(2)	8239	371	7.3	v2.0	546	7 [2]	7
*Pseudomonas aeruginosa*	PAO1	5647	559	1.6	v2.0	592	33	2
*Helicobacter pylori*	26695	1616	133	3.1	v2.0	256	8	5
*Bacillus anthracis*	Ames	5039	66	3.0	v3.0	75	4	4
*Bacillus cereus*	ATCC 14579	5231	283	2.4	v3.0	392	16	10
*Bacteroides thetaiotaomicron*	VPI-5482	4816	333	1.9	v3.0	353	19	4
*Campylobacter jejuni*	NCTC 11168	1572	152	12.5	v3.0	260	14	11
*Clostridium acetobutylicum*	ATCC 824	3778	377	2.4	v3.0	419	12	11
*Lactobacillus rhamnosus*	GG	2834	79	3.6	v3.0	158	3	2
*Methanococcus maripaludis*	S2	1722	364	1.5	v3.0	728	19	3
*Shigella flexneri*	301	4315	35	17.0	v3.0	38	3	3
*Sinorhizobium meliloti*	1021	6218	424	2.7	v3.0	713	20 [19]	10
*Streptococcus pneumoniae*	D39	1914	68	5.7	v3.0	136	12	2
*Thermus thermophilus*	HB8	2173	444	1.4	v3.0	480	6	3
*Yersinia pestis*	CO92	3979	36	6.1	v3.0	72	5	2

Rows of the table represent all the species and strains for which a gene expression compendium is hosted. Columns represent (from left to right): the species name, the strain used as reference genome for microarray probe to gene mapping and RNA-Seq read alignment, the total number of genes in the compendium, the total number of contrasts in the compendium, the percentage of missing values, the COLOMBOS version of the first inclusion of the respective species or strain, the total number of samples from which the compendium's contrasts are built, the total number of corresponding experiments on GEO and ArrayExpress (the latter indicated between square brackets) and the total number of platforms represented.

### Complete sample annotation

COLOMBOS sports an annotation system for condition contrast related meta-data which relies on a manually curated and controlled vocabulary. It is an essential information source that aids in the interpretation of gene expression patterns. As COLOMBOS condition contrasts represent comparisons between two samples (a ‘test’ sample compared to a ‘reference’ sample), in the past only condition properties which represented actual differences between the two samples were annotated. The major drawback of this approach is that it disregards what is shared between both samples: two contrasts could be annotated exactly the same regardless of the condition ‘background’ of their individual samples. For instance, when two contrasts had measured the exact same decrease in oxygen concentration, they would have been annotated identically. If one of the contrasts however had wild-type strains for both test and reference samples, and the other contrast had strains with a mutation in a gene important in aerobic respiration, this information would not be apparent from the contrast's annotation, while it is arguably an important factor to acknowledge. For this COLOMBOS update, we have fully overhauled the annotation system to instead work at the sample level (as opposed to the contrast level) and consequently hold the meta-information for both a contrast's samples’ experimental conditions, and not only the differences between them. When looking up a condition contrast in the COLOMBOS database, you will now be presented with the biological background (e.g. strains, medium, growth conditions) as well as the biological difference that results in the displayed expression behaviour.

## FUNCTIONALITY UPDATE

### Cross-species analysis

A completely new functionality in COLOMBOS v3.0 is the ability to work with all species simultaneously. The data from different organisms have been integrated on a higher level based on clusters of homologous genes (CHG) constructed with OrthoMCL v2.0.9 (12) using the default settings as applied to the protein sequences for the strains included in COLOMBOS v3.0. These CHGs can be thought of as the rows of an overarching expression matrix obtained by stitching together the individual compendia. Expression data for orthologous genes, i.e. genes assigned to the same CHG, are aligned across the respective species; species without a representative gene in a CHG can be thought of as having missing values. In case a CHG contains paralogous genes (multiple genes from the same species), their expression values are averaged. All data analysis tools included in COLOMBOS have been adapted to deal with these new cross-species compendia, so that this complex expression matrix can be queried and explored with the same flexibility as any single species. The cross-species comparison is not only a novelty for the identification of co-expressed gene sets across several species for e.g. evolutionary studies, but also has several advantages for the way compendia can be constructed. We can now build compendia for different strains and integrate them at the species level using homologue mappings. This has a clear advantage as, instead of using a single reference strain's genome to represent the species as was done before, we can now explicitly recognize genomic differences between strains and thus improve read alignment (RNA-seq) or probe to gene mapping (microarrays) to generate higher quality expression data. This concept has been used to improve our *Salmonella enterica sp. Typhimurium* compendium, where the original consisted of more or less equal parts of three different strains with minor differences in their genomic content.

### Analysis tools

Several changes have been made to web portal's suite of analysis tools and the RESTful web service and R API. These are mainly related to the query functionalities that actually make use of the expression values themselves (‘BLASTing with expression data’). While these previously looked solely for consistent co-expression, they are now capable of returning complex patterns of expression behaviour across sets of query genes (or conditions). For instance, in v2.0 the Quicksearch functionality would return, for a set of user defined genes, the contrasts where those genes behave in a similar and coherent way. These are not necessarily the most informative, or relevant, contrasts for the user, especially for larger gene sets for which co-expression behaviour might be rare and unrepresentative. By default the Quicksearch in v3.0 will visualize complex patterns of co-expression by running a biclustering on the returned module data, and will not necessarily return contrasts where the query input genes behave in the same way (although this functionality is still available in the Advanced search). Other improvements include various export functionalities so that COLOMBOS results can be easily imported in other widely used tools or databases (such as Cytoscape (13), BioCyc) for further downstream analysis.

## DISCUSSION AND FUTURE PLANS

COLOMBOS’ growths over the years have been a continuous effort towards better gene expression data integration and easier exploration and interpretation. Not only has the data more than doubled, but this last major update is another step in the direction of improving the strengths and eliminating the weaknesses of the previous version(s). The redesigned condition annotation system provides a more reliable interpretation of expression patterns with respect to the biological stimuli that are causing them. The new cross-species capabilities have the obvious advantage over the old system to be able to perform gene expression analyses on all species simultaneously, but also enable more accurate measurements mapping by separating different strains within the same species.

Keeping the compendia up-to-date, as well as expanding the scope by adding new organisms, is naturally our first priority. We generally select new species or strains based on data availability, but are always open to suggestions or requests from users who are interested in access to a gene expression compendium for a particular species. Further improvements and new functionalities that revolve around cross-species capabilities are planned for future versions. Flexibility regarding CHGs selection and composition, as well as new tools to empower users when dealing with complex CHGs are amongst the priorities. For instance, instead of being limited to pre-calculated, fixed CHGs for which homologues cannot be re-defined and that encompass all species in the compendia as is the case now, users will be able to define the settings to create CHGs for the species of their choice and consequently more dynamically integrate the data from the corresponding compendia. Updated tools will likewise enable a finer management of CHGs, unlike e.g. the current paralogues’ expression calculation that is averaged across all paralogues without the possibility for a different evaluation considering the variability amongst those paralogues, as well as give users the ability to compare expression derived measures, such as co-expression scores or networks, across species.
